# The Implications of the Developmental Origins of Health and Disease on Public Health Policy and Health Promotion in South Africa

**DOI:** 10.3390/healthcare4040083

**Published:** 2016-11-09

**Authors:** Sasiragha Priscilla Reddy, Anthony David Mbewu

**Affiliations:** 1 Human Sciences Research Council, University of the Western Cape, Private Bag X9182, Cape Town 8000, South Africa; 2 Department of Internal Medicine, Sefako Makgatho Health Sciences University, P.O. Box 60, Medunsa 0204, South Africa; tonymbewu@gmail.com

**Keywords:** developmental origins, health promotion, public health policy

## Abstract

The developmental origins of health and disease (DOHaD) hypothesis states that environmental influences in utero and in early life can determine health and disease in later life through the programming of genes and/or altered gene expression. The DOHaD is likely to have had an effect in South Africa during the fifty years of apartheid; and during the twenty years since the dawn of democracy in 1994. This has profound implications for public health and health promotion policies in South Africa, a country experiencing increased prevalence of noncommunicable diseases (NCDs) and risk factors and behaviours for NCDs due to rapid social and economic transition, and because of the DOHaD. Public health policy and health promotion interventions, such as those introduced by the South African Government over the past 20 years, were designed to improve the health of pregnant women (and their unborn children). They could in addition, through the DOHaD mechanism, reduce NCDs and their risk factors in their offspring in later life. The quality of public health data over the past 40 years in South Africa precludes the possibility of proving the DOHaD hypothesis in that context. Nevertheless, public health and health promotion policies need to be strengthened, if South Africa and other low and middle income countries (LMICs) are to avoid the very high prevalence of NCDs seen in Europe and North America in the 50 years following the Second World War, as a result of socio economic transition and the DOHaD.

## 1. Introduction

The developmental origins of health and disease (DOHaD) hypothesis states that environmental influences in utero and in early life can determine health and vulnerability to disease in later life through the programming of genes and/or altered gene expression [1,2,3]. David Barker showed that foetal malnutrition contributed to adult hypertension, obesity, and insulin resistance in the same individuals in later life [1]. Barker observed that the pattern of the relationship of deaths in infancy and adulthood reflected nutritional variations in early life in the same populations during adult life [2]. More recent studies have identified other diseases that can result from foetal over- or under-nutrition including immunological, mental health, and reproductive diseases [4,5,6].

Despite ample evidence of developmental plasticity and intergenerational influences in early life impacting on noncommunicable disease (NCD) in later life (especially in low socioeconomic groups), policy makers in high income countries (HIC) have been reluctant to implement health policy changes based on the DOHaD in order to reduce the burden of NCDs [3].

The DOHaD hypothesis could have even more profound implications for public health policy and health promotion in low and middle income countries (LMICs) such as South Africa. This is because the economic and humanitarian costs of NCDs are enormous and could destabilize the economies of such LMICs with their limited resources. Furthermore, recent data show that the risk markers for NCDs in LMICs become evident early in the process of socioeconomic improvement [3]. This is especially pertinent for South Africa, which is midway through a rapid social transition following the end of apartheid in 1994 [7]. This social and economic transition has resulted in an epidemiological transition [8] with increasing prevalence of NCDs and risk factors for NCDs [9,10]. However, the role of DOHaD in fueling this transition has not been fully explored. If the DOHaD phenomenon was functioning in South Africa during the apartheid years from 1948 to 1990, the result would have been an increase in NCDs and risk factors for NCDs in the period from 1990 to 2020 in children born to mothers who lived in severe poverty during those apartheid years. However, the quality of public health data over the past 60 years in South Africa precludes the possibility of proving that the DOHaD mechanism is responsible for some of the increase in NCDs and risk factors for NCDs of the past 20 years—such as the increasing prevalence of obesity, hypertension, and type II diabetes mellitus [11].

## 2. Mechanism of Developmental Origins of Health and Disease

Health promotion theory traditionally views the origins of health and disease arising firstly from the individual in terms of their behaviour and biology; and secondly from the ecological environment in which the individual operates. In this “nature or nurture” hypothesis of how genes and the environment cause disease, genes are non-modifiable risk factors, but environmental and behavioural factors are modifiable through public health programmes and health promotion interventions. The view has been that environmental and behavioural factors interact to alter body physiology and pathophysiology; whilst the genetic component of disease is immutable [1,2,12]; only undergoing change during the reassortment of genes in the germline at meiosis when the organism reproduces; or as a result of rare and random mutations.

More recently, however, evidence has gathered that there are other mechanisms at play in evolutionary biology [1,2,12] that can introduce phenotypic variation without substantially altering the genome. If the changes produced are reversible, the process is called accommodation. If the changes produced are irreversible, the process is called plasticity—for example, the genetic programming for the conservation of nutrients that occurs when the foetus is deprived of nutrients in utero. Other factors could also induce plasticity such as exposure to environmental chemicals, drugs, alcohol, infections, or stress during specific times of development, leading to functional changes in tissues, predisposing those tissues to diseases that manifest later in life [4]. Foetal alcohol syndrome is an example of such plasticity in the genome, and is highly prevalent in South Africa due to excessive alcohol consumption by mothers during pregnancy.

The third mechanism of variation in evolutionary biology is that of classical Darwinian natural selection, in which genes that programme for prolonged survival increase in frequency in a population; whilst those that programme for death before the age of fecundity, or programme for infertility, decline in prevalence in a population as organisms with such deleterious genes fail to reproduce, and do not pass those genes on to their progeny.

Environmental factors can then influence the epigenome—the non-coding alterations that affect DNA and thus influence gene expression—which can predispose these cells and tissues to disease/dysfunction across their lifespan: the so called “epigenetic” processes [4]. The plasticity of gene expression occurs partly because gene suppressors and promoters are sensitive to environmental and behavioural factors, and alter the expression of their target gene without changing its structure. This can be visualised using genome wide assays and gene chips. These chips display a colourful map of which genes have been upregulated and which have been downregulated, in images before and after an intervention. For example, when mice were fed a diet high in saturated fats to induce features of the metabolic syndrome, gene chips visually showed the upregulation of genes for the obesity-associated molecule leptin [13].

Health promotion interventions usually operate at the individual, biological, and behavioural levels, and secondly at the structural, societal level. These interventions are considered to be effective in preventing NCDs through altering body physiology. However, such interventions may alter gene expression in individuals, and alter the plasticity in genome expression in the foetus, according to the DHOaD mechanism.

## 3. Implications of the Developmental Origins of Disease Hypothesis for Low and Middle Income Countries

The DOHaD has profound implications for public health and health promotion policies in South Africa because the (black) majority population of South Africa before 1994 suffered from very high levels of poverty, undernutrition, and poor maternal and infant health. Since the advent of democracy in 1994, the South African Government has introduced massive social and health programmes, beginning with free primary health care for mothers and children—one of the first policy pronouncements of President Mandela when he came into office in May 1994 [7]. Social grant recipients increased from 2.7 million in 1994 to over 16 million by 2013—in a country with a population of 55 million. As a consequence, there was a significant reduction in headcount poverty, from 37 percent in 1993 to 8 percent in 2010 [7] (with most of the poor receiving social grants in one form or another); and average per capita incomes increased by 156 percent [14].

In the same two decades, the prevalence of risk factors for NCDs increased—with the notable exception of smoking, of which the prevalence halved from 33 percent to 16 percent between 1994 and 2014 as a result of extensive tobacco control legislation [15]. South Africa rapidly became the third most obese nation in the world with obesity rates of 20.1 percent and 10.6 percent in females and males respectively in 2014 [9]. The National South African Youth Risk Behaviour Surveys [10] documented an increase in the prevalence of overweight among male adolescents from 6.3 percent in 2002 to 11.0 percent in 2008; and among female adolescents, from 24.3 percent in 2002 to 29.0 percent in 2008. Obesity rates more than doubled among male adolescents from 1.6 percent in 2002 to 3.3 percent in 2008, and rose from 5.0 percent to 7.5 percent among female adolescents [10].

The prevalence of hypertension and diabetes mellitus have also increased in South Africa in the past 20 years [9,11]. Could it be that foetal programming in utero amongst impoverished pregnant black South Africans in the decades before 1994, resulted in phenotypes prone to NCDs in the offspring of these mothers during the past 20 years? This would be similar to the phenomenon that occurred in the Netherlands in the 1980s as a result of DOHaD amongst Dutch people born during the Dutch Famine of the Second World War [16].

## 4. Public Health Policy in South Africa

If such foetal programming has occurred amongst South Africans during the apartheid years from 1948 to 1994, then public health policy in South Africa should prepare for large increases in NCDs and their risk factors in the decades following 1994. Some public health policy changes have been made, including strict tobacco control policies [15]; and a Strategic Plan for NCDs [17] which sets clear interventions and targets for reductions in risk factors for NCDs (hypertension, smoking, obesity, lack of exercise, and poor diet) over the years leading up to 2022. These interventions will reduce NCDs directly by acting upon the risk factors for NCDs. They may also reduce NCDs and risk factors for NCDs in the future by improving the health of pregnant women who are susceptible to the DOHaD mechanism, by for example reducing smoking during pregnancy.

Other public social and health policy interventions that may reduce NCDs in the future in South Africa through the DOHaD mechanisms include widespread social grants for mothers and children; and interventions to improve the health of pregnant women. Such interventions may be partly responsible for the improvement in the “institutional” maternal mortality rate (iMMR), which in 2008–2010 had reached a very high peak of 176.22 maternal deaths per 100,000 live births. By the 2011–2013 triennium, the iMMR had declined to 154.06 per 100,000 live births [18]. These interventions may also reduce NCDs in the offspring of these mothers in later life through the DOHaD mechanism.

Another public health intervention that, through the DOHaD mechanism, may reduce NCDs in the future in South Africa is the “Operational Plan for Comprehensive HIV and AIDS Care, Management and Treatment for South Africa”. This was launched in 2003 at the height of the HIV and AIDS epidemic; and over 3.7 million people have had access to treatment since then, including young women who are pregnant or are soon to become pregnant [19]. From the outset, it was recognised that many people (including pregnant women) living with HIV and AIDS were food insecure, and their HIV infection represented a highly catabolic state, that would be exacerbated once they started treatment [19]. Consequently, the policy now is to offer all food parcels and multivitamins in addition to their antiretroviral therapy. Nearly 30 percent of the one million women who give birth each year in South Africa are HIV positive [20]. The DOHaD hypothesis would predict that these 300,000 HIV positive women who give birth each year would be particularly susceptible to foetal nutrient deprivation, resulting in NCDs and risk factors for NCDs in their offspring during adulthood. Their health has been transformed by the introduction of free antiretroviral therapy for all such pregnant women as soon as they are diagnosed as HIV positive at antenatal screening; as well as food supplementation and multivitamins [20]. These interventions are especially important in early gestation, the period in which the foetus is most susceptible to the effects of DOHaD [12].

Lastly, the government should continue with the progress that has been made in the past 20 years in vastly improving the economic situations and living environments of all South African citizens, especially pregnant women because of the DOHaD hypothesis. For example, living environments have been continuously upgraded through the construction of 2.8 million houses over the past 20 years [7], but many more still need to be built.

Whilst no DOHaD interventions have specifically been made in South Africa as of yet, many public health and social policies and health promotion interventions [7] that are likely to impact on DOHaD in pregnancy and early life have been made over the past 22 years. These include:

- tobacco control legislation
- free primary health care for pregnant women and infants, including antenatal care and the expanded programme of immunisation for infants
- free and compulsory primary and secondary school education
- prevention of HIV transmission to the foetus in pregnancy and labour through antiretroviral therapy
- antiretroviral provision to HIV positive pregnant women (regardless of CD4+ count)
- food supplements and multivitamins for HIV positive pregnant women
- improved housing, water, sanitation, and household electrification
- social grants for pregnant women

None of these interventions are likely to abolish the increasing levels of diabetes, hypertension, and heart disease that South Africa is likely to experience over the next 20 years (partly as a result of foetal programming over the past 40 years)—but they should blunt the rise in the prevalence of NCDs and risk factors for NCDs. Already there are signs that South Africa may avoid the very high levels of heart disease that the USA and Europe experienced in the 1970s, as mortality from ischaemic heart disease seems to have risen more slowly over the past 20 years than had been expected (though some of this may be due to under reporting) [21].

## 5. Conclusions

The DOHaD hypothesis has important public health and health promotion policy implications for South Africa. This is because South Africa is midway through a rapid social transition following the end of the apartheid era in 1994 [7]. This social transition has seen the prevalence of severe poverty decline from 37 percent to 8 percent, and average per capita incomes increase by 156 percent [8]. South Africans born between 20 and 40 years ago to impoverished mothers could therefore experience increased prevalence of NCDs and risk factors for NCDs in their adult years due to the DOHaD process operating in South Africa.

The South African Government has undertaken public health policies and health promotion interventions over the past 22 years to improve the health of pregnant women (and their unborn children), and to reduce NCDs and the risk factors for NCDs in children and adults [7]. The most notable of these has been a raft of tobacco control legislation since the first democratic election in 1994 [15]. This has resulted in a decline in smoking prevalence from 33 percent to 17 percent in adults, with similar changes in school learners. This will have a direct impact on NCDs in adults in the long term, but also through reducing the exposure of the foetus in pregnant women in early gestation to chemicals derived from tobacco smoke.

Mortality from ischaemic heart disease remains at moderate levels in South Africa (though this may be due to underreporting) [21], but obesity [9,10] and diabetes levels are increasing [11], and hypertension and stroke are highly prevalent [9]. This increase in NCDs and the risk factors for NCDs may have been partly due to the DOHaD process in pregnant women during the deprivations experienced in the apartheid era from 1949 to 1990. Maternal and infant mortality levels are declining from the very high peak seen in 2008 [18], which may relate to socioeconomic changes as well the introduction of antiretroviral drugs to reduce HIV transmission from mother to child; and to the delivery of lifelong antiretroviral treatment for all women diagnosed HIV positive during pregnancy [19,20]. HIV positive women on antiretroviral treatment are also offered food supplementation and multivitamins. All these interventions may be important if the DOHaD mechanism is operative in South Africa, as such interventions could well affect foetal programming, and consequently NCDs and the risk factors for NCDs in the mothers’ offspring in the 30–50 years following birth.

Therefore, in South Africa, NCD prevention begins with improved nutrition and reduced exposures to environmental chemicals during development [4]. For many years, the focus on the processes that influence the risk of NCD has been largely on genetic or adult lifestyle factors, to the exclusion of this third, critical component, namely development [4,22]. For many diseases, it is clear that a new focus on the timing of disease prevention is needed [4]. The lifespan approach to prevention and health promotion should not only encompass the decades from birth to death, but should also include the 9 months spent in utero by improving maternal health. Early interventions during the prenatal period and the first few postnatal years, when tissues are forming and the epigenetic system is most sensitive to environmental insults, have the potential to lead to improved lifelong health through the DOHaD mechanism [4]. The targets for reducing the risk of disease across lifespans and generations are the pregnant mother, their infants, and young children through puberty [4].

Altered gene expression and epigenetic changes caused by environmental influences in utero and during childhood and adulthood [4] can explain the profound effects of health promotion interventions on health and disease without belabouring the false and reductionist dichotomy of “nature versus nurture”, or “gene versus environment” [22]. This may be especially important in LMICs, such as South Africa, that are undergoing rapid social epidemiological transitions. Unfortunately, the quality of public health data over the past 40 years in South Africa precludes the possibility of proving the DOHaD hypothesis in this country.

Whilst no DOHaD interventions have specifically been made in South Africa as of yet, the public health and social policies and health promotion interventions in South Africa [7] that are likely to impact on DOHaD in pregnancy and early life have been listed above. These are likely to be effective in HIV negative pregnant women, but especially effective amongst the 30% of pregnant women who are HIV positive, as their unborn children are exposed to a more deleterious environment in utero due to the stress of their mothers’ HIV disease. Indeed, the decline in maternal and infant mortality seen since its peak in 2008 is likely to be at least in part caused by the interventions mentioned above.

Such public health policy and health promotion policies for pregnant women (because of the DHOaD) and the general population throughout the course of life need to be strengthened, if South Africa and other LMICs are to avoid the very high prevalence of NCDs seen in Europe and North America in the 50 years following the Second World War.

## Abbreviations

The following abbreviations are used in this manuscript:

DOHaD	The developmental origins of health and disease
LMIC	low and middle income countries
NCD	noncommunicable disease
LDL	low-density lipoprotein
DNA	deoxyribonucleic acid
RNA	ribonucleic acid
HIV	human immunodeficiency virus
AIDS	acquired immune deficiency syndrome
iMMR	institutional maternal mortality rate
